# Detection of Network Motif Based on a Novel Graph Canonization Algorithm from Transcriptional Regulation Networks

**DOI:** 10.3390/molecules22122194

**Published:** 2017-12-10

**Authors:** Jialu Hu, Xuequn Shang

**Affiliations:** 1 School of Computer Science, Northwestern Polytechnical University, West Youyi Road 127, Xi’an 710072, China; shang@nwpu.edu.cn; 2 Centre for Multidisciplinary Convergence Computing, School of Computer Science, Northwestern Polytechnical University, Dong Xiang Road 1, Xi’an 710129, China

**Keywords:** network motif, algorithms, graph canonization

## Abstract

Network motifs are patterns of complex networks occurring significantly more frequently than those in random networks. They have been considered as fundamental building blocks of complex networks. Therefore, the detection of network motifs in transcriptional regulation networks is a crucial step in understanding the mechanism of transcriptional regulation and network evolution. The search for network motifs is similar to solving subgraph searching problems, which has proven to be NP-complete. To quickly and effectively count subgraphs of a large biological network, we propose a novel graph canonization algorithm based on resolving sets. This method has been implemented in a command line interface (CLI) program sgip using the SeqAn library. Comparing to Babai’s algorithm, this approach has a tighter complexity bound, $o(\exp\left(\sqrt{n}\log^{2}n+4\log n\right))$, on strongly regular graphs. Results on several simulated datasets and transcriptional regulation networks indicate that sgip outperforms nauty on many graph cases. The source code of sgip is freely accessible in https://github.com/seqan/seqan/tree/master/apps/sgip and the binary code in http://packages.seqan.de/sgip/.

## 1. Introduction

With the advent of high-throughput technologies in biology, the task of obtaining genetics and transcriptional information from specific tissues has become easier and more cost-effective. For example, the yeast two-hybrid (Y2H) system, Chromatin Immunoprecipitation Sequencing (ChIP-seq), and co-immunoprecipitation (coIP) coupled to mass spectrometry allow us to screen molecular interactions of a cell on a large scale. This achievement shed light on the research of understanding the underlying transcriptional regulation and network evolution. It can help us in unraveling the encrypted messages encoded in the structure and topology of protein-protein interaction (PPI) networks. To be analogous with sequence motif, network motif was proposed to characterize different types of complex networks. Network motifs are patterns of interactions occurring in a complex network at numbers that are significantly higher than those in randomized networks. They are considered as basic building blocks which play key roles in processing cellular signals in transcriptional regulatory networks. Currently, several major network motifs have been found in transcriptional regulatory networks, which include feed-forward loop (FFL), single input module (SIM), and dense overlapping regulons (DORs) [1]. The detection of a motif in a PPI network reveals that motif networks substantially influence the evolution conservation.

Many analysis tools have been developed for detecting network motifs in the last decade, including FANMOD [2], mfinder [3], MAVisto [4], etc. There are three major subproblems in the detection: (1) generating an ensemble of proper random networks; (2) exhaustively enumerating all possible subgraphs in a real network and randomly generated networks; (3) grouping these subgraphs into different categories by the topological structure ; (4) calculating the statistical significance of each graph pattern. Our work mainly focuses on the third subproblem, which is a classical graph canonization problem. Graph canonization is a fundamental problem in theoretical and practical computer science. Its theoretical importance is derived from the relationship with the graph isomorphism problem (GIP). In practice, graph canonization algorithms have many applications in data mining [5,6] and pattern recognition [7,8]. Many of these algorithms were also used in chemistry [9,10,11]. Therefore, we are motivated to close the gap of computational complexity in the graph canonization problem and develop efficient programs to solve practical problems.

For the graph canonization problem, the complexity of the best known algorithms are moderately exponential $\exp(n^{\frac{1}{2}+o\left(1\right)})$ for general graphs, sub-exponential time $n^{n\log n}$ for tournaments [12], polynomial time for bounded valence graphs [13], and $o(\exp\left(2n^{\frac{1}{2}}\log^{2}n\right))$ for strongly regular graphs [14]. An algorithm proposed in [15] can canonically label *d*-regular graphs in linear average time cdn. An improved work [16] was done for strongly regular graphs, which can solve the graph isomorphism problem in time $n^{O(n^{\frac{1}{3}}\log n)}$. A more recent work [17] stated that the graph isomorphism for hypergraphs of rank *k* can be solved in moderately exponential time $\exp(\tilde{O}\left(k^{2}\sqrt{n}\right))$. In addition to all these theoretical results, practical algorithms were also developed and applied in various applications. One of the most notable and widely used tools is the nauty package [18], which employs a canonical labeling approach to compute automorphism groups. A comprehensive introduction of Mckay’s algorithm was described in [19]. However, this algorithm also suffered from an exponential running time on a family of graphs constructed by Miyazaki [20].

Backtracking algorithms are also used to determine whether two general graphs are isomorphic by adopting a heuristic function in the search tree, such as SD [21], VF [22] and VF2 [23]. A comparison analysis of the performance of VF2 against VF, nauty, SD, and Ullman [24] was presented in [25]. A new symmetry-detection tool, saucy [26] was developed and tested on large structured graphs generated by CNF formulas, which can outperform nauty by several orders of magnitude. A more recent work, bliss [27], relying on the individualization and refinement scheme in the framework of backtracking, can outperform existing tools in most cases.

Inspired by the concept of the distinguishing set presented in [14], we first introduce the *resolving set* on graphs to solve the graph canonization problem based on backtracking approaches. In this paper, our aim is to design a canonical labeling algorithm that can extend Babai’s algorithm to general cases and make it possible to use in practice. Furthermore, we attempt to close the remaining complexity gap of Babai’s algorithm [14] on strongly regular graphs with λ=μ+1. It is noted that our aim is to solve the problem in most graph cases, not in the worst case. As far as we know, all the existing algorithms have exponential running time on some types of particularly designed graphs.

Our contribution in this work lies in the following three aspects: (1) we propose a novel algorithm, sgip, for solving the third subproblem in the detection of network motifs; (2) compared to previous algorithms, our approach is characterized by a tighter complexity bound of graph canonization problem; (3) we implemented this novel algorithm in the distribution of SeqAn library [28], which is comparable to the notable package nauty in practice.

## 2. Preliminary and Definitions

### 2.1. Subproblem of Grouping Subgraphs

Graph canonization is to find a canonical form for a given graph, which is still an open problem that is neither known to be a polynomial complete nor an NP-complete problem [29]. To group a set of subgraphs into different categories, it is necessary to find a canonical label for each category. Given two graphs $G=(V_{1},E_{1})$ and $H=(V_{2},E_{2})$, graph canonization is to find a canonical labeling function 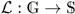 such that *H* is isomorphic to *G* if and only if L(G)=L(H), where 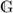 represents a set of graphs and 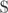 indicates a collection of strings. Obviously, the graph canonization problem (GCP) is at least as hard as the graph isomorphic problem.

### 2.2. Definitions

The resolving set concept was first introduced in the literature [30] under the term *locating set*. It is analogous to the distinguishing set in Babai’s straightforward algorithm. Assuming each node has a unique position in a given graph, the position can be simply determined by a set of vertices in the graph. This set of vertices is then referred to as a resolving set of this graph. Let d(u,v) be the *distance* (i.e. number of edges in the *shortest path*) between any two vertices u,v∈V(G).

**Definition** **1.** Given an ordered subset of vertices $W=(w_{1},w_{2},\cdots ,w_{s})$ of V(G), the s-tuple $r\left(v|W\right)=(d\left(v,w_{1}\right),d\left(v,w_{2}\right),\ldots ,d\left(v,w_{s}\right))$ was referred to as the metric representation of v with respect to W. W is called a resolving set if and only if r(v|W)≠r(u|W) for distinct v and u.

**Definition** **2.** *The smallest resolving set of a graph G is called a minimum resolving set or* metric basis *of G, denoted as MRS.*

**Figure 1:**
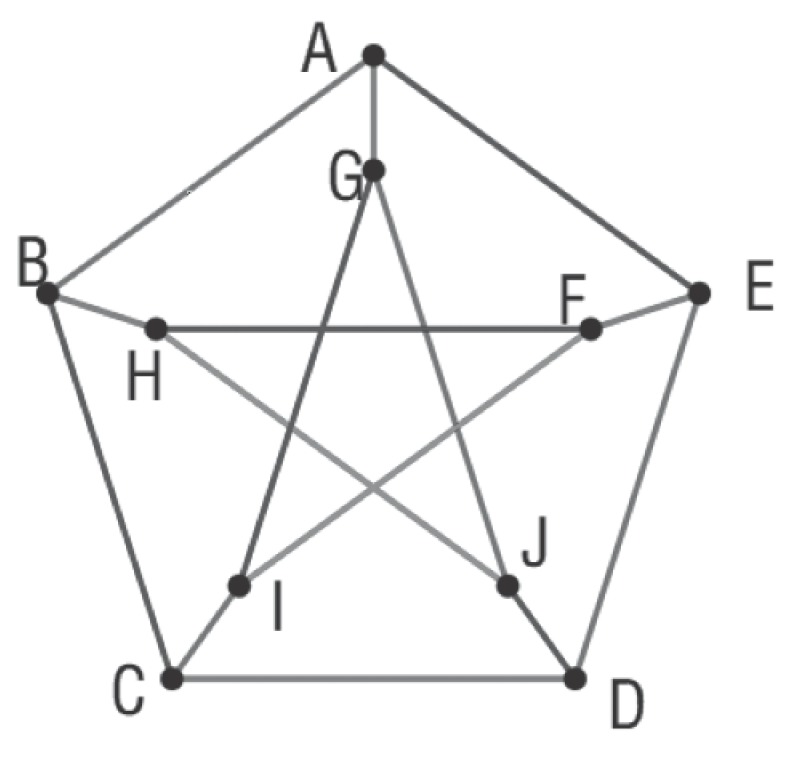
Metric dimension of the Petersen graph. Checking each node, we find that W=(A,D,I) is one possible resolving set, and no smaller resolving set exists. Hence, the metric dimension of the Petersen graph is μ(g)=3.

From the definition, we known that a MRS gives a *unique metric representation* for each vertex in *G*. A graph can be seen as an expansion of *W* in multidimensional space, and all vertices of *G* have their own unique coordinate positions in the expansion space. The *metric dimension* of *G*, denoted as μ(G), is the cardinality of the minimum resolving set of *G*. For instance, Petersen graph shown in Figure 1 can be determined by *MRS*(A,D,I), and its metric dimension is μ(G)=3. Each vertex has a unique metric representation with respect to *metric basis*(A,D,I). For example, r(B|W)=(1,2,2), r(E|W)=(1,1,2) and r(C|W)=(2,1,1).

**Definition** **3.** A graph G is said to be k-regular if ∀v∈V(G), the degree of v is k. A k-regular graph G is called a strongly regular graph with parameters (n,k,λ,μ) if all the following conditions hold: (1) G is neither complete nor empty; (2) any two adjacent vertices of G have λ common neighbors; (3) any two nonadjacent vertices of G have μ common neighbors.

## 3. Methods

To efficiently distinguish all subgraphs enumerated from biological networks, we propose a novel canonical labeling function based on minimum resovling sets. By definition, each minimum resolving set determines an ordered set of graph nodes. Given a graph, we exhaustively search all possible minimum resolving sets and assign a lexicographical leader as its canonical form.

### 3.1. Canonical Labeling Function

Let $W=(w_{1},w_{2},\cdots ,w_{s})$ be a resolving set for an input graph Γ=(VΓ,E). As shown in the Definition 1, any pair of v,u∈VΓ has distinctive metric representation, r(v|W)≠r(u|W). Therefore, by defining that r(v|W)≺ r(u|W) if there exists an integer *j* such that $d\left(u,w_{j}\right)<d\left(v,w_{j}\right)$ and $d\left(u,w_{i}\right)=d\left(v,w_{i}\right)$ for all integer i<j, we can sort all the objects in VΓ by the metric representation taking *W* into account. So, each resolving set uniquely determines an adjacent matrix $Adj_{W}\left(\Gamma \right)$. Then, we give the definition that a canonical label is assigned by the lexicographical leader of all the adjacent matrix yielded by all possible resolving sets. Mathematically, the canonical labeling function can be written in

$$L\left(\Gamma \right)=\min_{⪯lex}\left\{Adj_{W}\left(\Gamma \right)|W\in \Omega \right\}$$

where Ω indicates the collection of minimum resolving sets for graph Γ. So, in order to compute the canonical label, an exhaustive search should be performed on a list of all potential resolving sets. Then, feasible object combinations should be selected and the optimal one should be found, corresponding to the least lexicographical adjacent matrix. Obviously, the complexity of our algorithm only depends on μ(Γ). Supposing |W|=k, we can easily draw a conclusion that all k! permutations of *W* are resolving sets if *W* is a resolving set through Definition 1. Therefore, if there are *C* different feasible combinations of vertices in all which yield *minimum resolving sets*, the number of minimum resolving sets is |Ω|=C·μ(Γ)!. Let Γ be a strongly regular graph which is neither the union of disjoint complete graph nor the complement of such a graph. Since the distinguishing set is equivalent to the resolving set for strongly regular graphs, the metric dimension μ(Γ) is bound to $\lfloor 2\sqrt{n}\log n\rfloor -3$ according to Babai’s complexity theory. However, this exhaustive approach also suffers in a large computation for graphs with high metric dimension, such as complete graphs (μ(Γ)=n−1) and stars (μ(Γ)=n−2). Therefore, it is impractical to use on general cases.

### 3.2. Extended Algorithm on General Cases

In order to find a canonical label for these graphs of high dimension, we introduced the concept of *parity nodes*, also called *twin vertices* [31], to prevent redundant computation, because some distinct resolving sets can deduce to identical adjacent matrix. For simplicity, notations are defined as follows on general graphs. For the undirected case, N(v) refers to neighborhoods of node *v*, δ(v) indicates degree of node *v*. For the directed case, we denoted with $N^{+}\left(v\right)$, $N^{-}\left(v\right)$, and N(v), respectively, as in, out, and total neighborhoods of *v*; analogously with $\delta^{+}\left(v\right)$, $\delta^{-}\left(v\right)$, and δ(v), we denote it as in, out, and total degree of *v*.

**Definition** **4.** *For undirected graph Γ=(VΓ,E), two vertices u,v∈VΓ are called parity nodes, and denoted as u↔v, iff it holds*

N(u)∖v=N(v)∖u;

*otherwise, they are non-parity nodes, denoted as u↮v.*

**Definition** **5.** *For directed graph Γ=(VΓ,E), two vertices u,v∈VΓ are called parity nodes and denoted as u↔v iff all of the following conditions are satisfied*

$$\left\{\begin{array}{c} \delta^{+}\left(u\right)=\delta^{+}\left(v\right)\\ \delta^{-}\left(u\right)=\delta^{-}\left(v\right)\\ N^{+}\left(u\right)\setminus v=N^{+}\left(v\right)\setminus u\\ N^{-}\left(u\right)\setminus v=N^{-}\left(v\right)\setminus u \end{array}\right.$$

*otherwise they are non-parity nodes, denoted as u↮v.*

Parity nodes define an *equivalence relation* on VΓ. The *reflexive* property can be easily verified. From the definition of parity nodes, we can easily figure out the symmetric and transitive properties: (1) for any two vertices u,v∈VΓ, if u↔v, then (u,v)∈E⇔(v,u)∈E; (2) for any three vertices u,v,w∈VΓ, if u↔v and v↔w then u↔w. The equivalence relation defined by parity nodes is an invariant for all types of graphs. So, the node set VΓ can be classified into a set of *equivalent sets* by parity nodes. The appearance of parity nodes is a major cause of high metric dimension in general graphs, since nodes from one equivalent set cannot be distinguished by any other nodes through metric representation except themselves. For instance, it is clear that all nodes of a complete graph $K_{q}$ are in one equivalent set. So, $\mu (K_{q})=q-1$ that makes the search task intractable. To extend the former algorithm working on general cases, we propose the concept of the *improved resolving set* that can assign canonical label for input graphs with a smaller metric dimension than the *resolving set*.

For a graph Γ=(VΓ,E), suppose VS is a subset of VΓ such that for any equivalent set *S* in VΓ it implies |VS∩S|=1, *W* which is a subset of VS is called *advanced resolving set* if any two elements $v_{i},v_{j}\in VS$, it satisfies $r\left(v_{i}|W\right)\neq r\left(v_{j}|W\right)$. Those with minimum cardinality are called *minimum advanced resolving sets*, and the minimum cardinality is called the *advanced metric dimension* of Γ, denoted as ν(Γ).

According to the definition, the *improved resolving set* only takes one node from each equivalent set into account. Obviously, the *advance metric dimension* is smaller than the *metric dimension*. Then, it concludes that the *improved resolving set* determines a unique order for a part of vertices in VΓ. However, one may ask the question: does it work for the whole graph? In other words, how does it determine a canonical label for a given graph? This question will be answered in two steps. Before all that, it is notable that given a graph Γ with *n* vertices, $P_{n}$ indicates the set of permutations and $\forall \sigma \in P_{n}$, $Adj_{\sigma }\left(\Gamma \right)$ represents the adjacent matrix of graph Γ.

For a graph Γ and $\forall \sigma \in P_{n}$, if u,v∈VΓ and u↔v, then by inverting the order of *u* and *v* in σ we obtain a new permutation τ such that $Adj_{\sigma }\left(\Gamma \right)=Adj_{\tau }\left(\Gamma \right)$. Let $\sigma_{x}=v$, $\sigma_{y}=u$, $A=Adj_{\sigma }\left(\Gamma \right)$, and $B=Adj_{\tau }\left(\Gamma \right)$. Then, $\tau_{x}=u$ and $\tau_{y}=v$, and there must exist a permuted matrix *P*, such that $A=P^{-1}BP$. By Definition 5, we know that $N^{+}\left(v\right)\setminus u=N^{+}\left(u\right)\setminus v$. This implies that A(x,j)=A(y,j), j∉{x,y}. Similarly, $N^{-}\left(v\right)\setminus u=N^{-}\left(u\right)\setminus v$ implies that A(j,x)=A(j,y), j∉{x,y}. Additionally, v↔u implies A(x,y)=A(y,x), and clearly, A(x,x)=A(y,y)=0, hence it follows $A=PAP^{-1}=B$.

Through the theorem stated above, the *advanced resolving set* is able to determine a canonical label for input graphs. All of the permutations within the equivalent set yield the same adjacent matrix. Hence, our algorithm for assigning a canonical label for a given graph Γ can be done in the following four steps: (1) determine equivalent sets over VΓ; (2) calculate ν(Γ) on the graph Γ; (3) give a brute force search over a set of non-equivalent nodes; (4) choose the optimal one which has the lexicographical leader. This improved algorithm provides a better performance on these graphs with rich equivalent sets. The *advanced resolving set* enables our algorithm to work on many types of graphs.

### 3.3. Complexity Analysis

As stated above, the complexity of the straightforward algorithm and the extended algorithm primarily depend on *metric dimension* and *advanced metric dimension*, respectively. According to Babai’s theorem [14], the complexity of his algorithm on strongly regular graphs is bounded by $o(\exp\left(2\sqrt{n}\log^{2}n\right))$. Here, we proposed a statistical model revealing that a tighter bound of complexity exists on strongly regular graph with μ=λ+1.

Supposing ν(Γ)=s for a general graph Γ, it takes time $O(n^{2})$ to compute equivalent sets, $O(n^{2})$ to calculate the distance matrix, and $O(sn^{2})$ time to test whether it is a resolving set. So, the complexity of our algorithm is simply bounded by $o(n^{s+3})$. Since $\mu \left(\Gamma \right)\leq \sqrt{n-1}\log n+1$ for strongly regular graphs with μ=λ+1, we set a new tighter bound $o(\exp\left(\sqrt{n-1}\log^{2}n+4\log n\right))$ in recognizing this kind of particularly designed graphs.

## 4. Results and Discussion

All of our experiments were carried out on a Linux PC with 2.2 GHz CPU, 4 G memory. The algorithm was implemented in a package named sgip in the distribution of the C++ library SeqAn. The source code of sgip is freely accessible at https://github.com/seqan/seqan/tree/master/apps/sgip and the binary code at http://packages.seqan.de/sgip/.

### 4.1. Tests on Simulated Data

To test the performance, our algorithm and the nauty algorithm were tested on a benchmark database [32] containing 72,800 couples of simple graphs, of which 18,200 couples are isomorphic graphs and 54,600 couples have subgraph isomorphism mapping among them. These graphs are in several different categories, which include randomly connected graphs, regular meshes, bounded valence graphs, irregular meshes, and irregular bounded valence graphs. In addition, 3400 new couples of isomorphic random graphs were generated.

**Figure 2:**
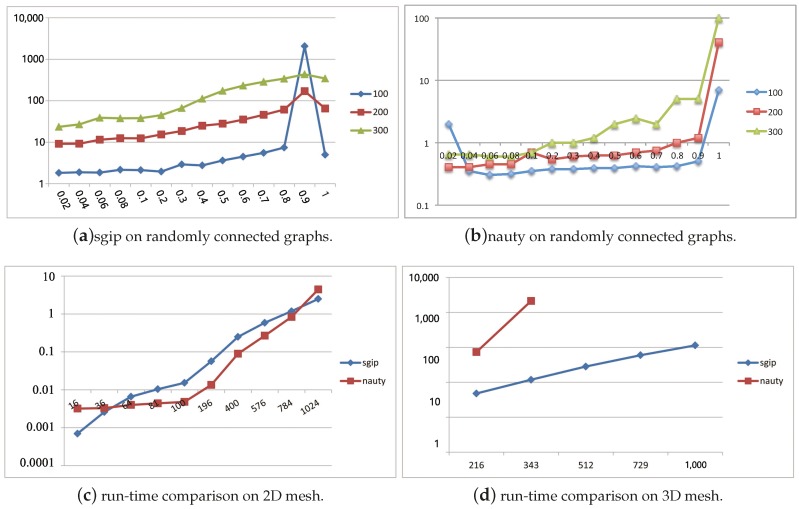

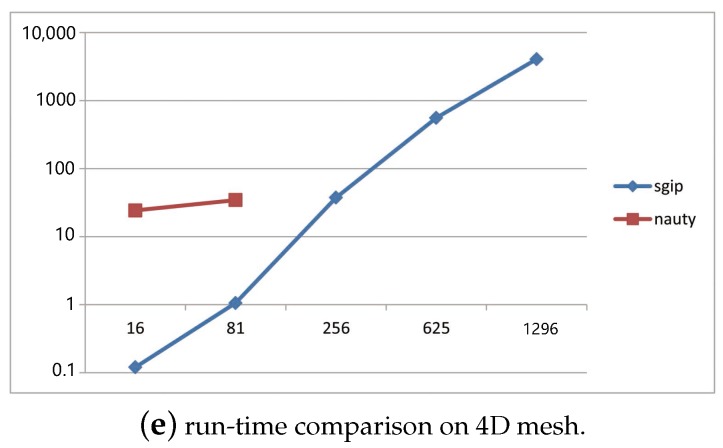
Performance comparison of sgip and nauty on different types of graph. Each experiment was tested on 100 couples of isomorphic graphs. (**a**,**b**) Horizontal axis represents the density of tested graphs (density is the ratio of the number of existing directed edges and *n*(*n* − 1)), vertical axis represents the running time of the responding tool. (**c**,**d**,**e**) Horizontal axis represents the number of vertices of the tested graphs, and the vertical axis is run-time on mesh graphs.

To provide a comparison of sgip with nauty, Figure 2 consists of five plots sketching the running time of two packages over various kinds of graphs. Average running time was estimated by sampling 100 couples of general graphs for each density. Figure 2a,b give an overview of the growing trends of sgip and nauty with respect to a density series. It is true that the running time of the two tools basically depends on both the density and size (number of nodes) of given graphs. It is worth noting that sgip took approximately 20 min to figure it out on graphs with 100 nodes and density of 0.9. We think that this was caused by some special graphs of highly advanced metric dimension. Overall, all kinds of tested general graphs could be distinguished in a reasonably short time, including both sparse and dense graphs, in spite of a slightly longer running time than that of nauty in general usage. However, there are also many solid pieces of evidences showing that sgip outperforms nauty in some other cases. For instance, Figure 2c–e depicts plots of running time cost on mesh graphs versus graph size. It is shown that nauty had a dramatic increase when the size of the mesh graphs got larger, and it became impractical to use when the size of the 3D mesh graphs exceeded a certain limit (around 300). More seriously, on 4D mesh graphs, it was unable to compute when the size was only above 81. In contrast, sgip was more than 100 times faster than nauty on 3D mesh graphs. The advantage of sgip became increasingly conspicuous with the growth of size and dimension on mesh graphs. Apparently, sgip is rather feasible for use in multi-dimensional mesh graphs.

### 4.2. Tests on Transcriptional Regulation Networks

To apply our algorithm in the detection of network motifs from transcriptional regulation networks, we employed the *ESU* algorithm to search for all possible subgraphs in PPI networks. In the following step, the program sgip was adapted to solve the subproblem of grouping subgraphs into different categories. Our approach was performed on transcriptional regulation networks of *Saccharomyces cerevisiae* and *Escherichia coli*. One thousand random graphs were generated for each species by randomly switching any two interactions of the real network 10∗|E| times, which can guarantee the same degree distribution with the real network. Finally, *p*-value was used to measure the statistical significance of each pattern. As shown in Table 1, we found three types of network motif which frequently occur in real biological networks. These patterns are feed-forward loop, single input module, and pairs of operons controlled by the same two transcriptional factors. All of their *p*-value scores were less than 1 × 10^−13^.These results indicate the practicability of our algorithm in the detection of network motifs in transcriptional regulation networks.

## 5. Conclusions

In this paper, we proposed a novel graph canonization algorithm sgip based on resolving sets to count all possible subgraphs of a large transcriptional regulation network. It has been proven that there exists a tighter bound of metric dimension on strongly regular graphs with μ=λ+1. The algorithm was implemented in the distribution of C++ library SeqAn. To test the performance, both nauty and sgip were performed on the same benchmark datasets with identical computational resources. The result shows that sgip is efficient and practical to use in most general cases and outperformed nauty in some particular designed graph structures. Applied in transcriptional regulation networks, our approach successfully found three typical network motifs which were significantly more frequent than those in randomized networks. This proves the efficiency of sgip in the detection of network motifs from large biological networks. Graph canonization problems are widely used in many other scientific and engineering fields, such as pattern recognition, chemical structure, etc. Hopefully, the practicability of our algorithm can benefit more researchers in their work and studies.

## Figures and Tables

**Table 1:** Statistically significant patterns appearing in transcriptional regulation networks. Pattern A refers to a coherent feed-forward loop whose connections are x→y→z and x→z. Pattern B refers to these subgraphs with single input module (>10 nodes). Pattern C refers to pairs of operons controlled by the same two transcription factors.

Patterns	Appearances in Real Network		Appearances in Randomized Network		*p*-Value	
	Yeast	*E. coli*	Yeast	*E. coli*	Yeast	*E. coli*
A	65	34	10.2 ± 5	4.5 ± 2	0	0
B	122	79	33.5 ± 12	30 ± 6	8.2 × 10^−14^	1.1 × 10^−16^
C	396	203	108 ± 29	55 ± 10	0	0
